# MACE-Seq-based coding RNA and TrueQuant-based small RNA profile in breast cancer: tumor-suppressive *miRNA-1275* identified as a novel marker

**DOI:** 10.1186/s12885-021-08218-4

**Published:** 2021-04-29

**Authors:** Sevan Omer Majed, Suhad Asad Mustafa

**Affiliations:** 1 Biology Department, College of Education, Salahaddin University-Erbil, Erbil, Iraq; 2 Research Center, Molecular Genetics lab, Salahaddin University-Erbil, Erbil, Iraq

**Keywords:** Breast cancer, miRNA, Small RNA and gene expression, *miR-1275* and its target genes, Pathogenesis, And tumor suppressor

## Abstract

**Introduction:**

Disruption of cellular processes in the breast by abnormally expressed miRNA is characterized to develop cancer. We aimed to identify the differential expression of small RNAs (sRNAs) and mRNAs in formalin-fixed paraffin-embedded (FFPE) tissue of the breast cancer (BC) and normal adjacent tissue (NAT). Another aim is to determine the differential expression of *miR-1275* as a novel biomarker for BC and also identify its target genes.

**Methods:**

TrueQuant method for analysis of sRNA expression and MACE-sequencing method for analysis of gene expression were used analyzing. The RT-qPCR technique was used to confirm *miR-1275* down expression. Target genes of *miR-1275* were computationally identified using target prediction sites and also the expression level of them was experimentally determined among the expressed genes.

**Results:**

TrueQuant findings showed that 1400 sRNAs were differentially expressed in the FFPE tissue of two Kurdish cases with BC, as compared to NAT. Among the sRNAs, 29 small RNAs were shown to be significantly downregulated in BC cells. The RT-qPCR results confirmed that *miR-1275* was significantly down-expressed in 20 Kurdish cases with BC compared to NAT. However, Overall survival (OS) analysis revealed that the correlation between the expression level of *miR-1275* and clinical significance was highly corrected in cases with BC (OS rate: *P* = 0.0401). The MACE-seq results revealed that 26,843 genes were differentially expressed in the BC tissue compared to NAT, but 7041 genes were displayed in a scatter plot. Furthermore, putative target genes (*DVL3*, *PPP2R2D*, *THSD4*, *CREB1*, *SYT7*, and *PRKACA*) were computationally identified as direct targets of *miR-1275* in several target predicted sites. The MACE-seq results revealed that the expression level of these targets was increased in BC tissue compared to NAT. The level of these targets was negatively associated with *miR-1275* expression. Finally, the role of down-regulated *miR-1275* on its targets in biological mechanisms of BC cells was identified; including cell growth, proliferation, movement, invasion, metastasis, and apoptosis.

**Conclusion:**

Down-expressed *miR-1275*, a tumor suppressor, is a novel biomarker for early detection of BC. *DVL3*, *PPP2R2D*, *THSD4*, *CREB1*, *SYT7*, and *PRKACA* are newly identified to be targeted by *miR-1275*.

## Introduction

Malignant breast tumor is a prominent type of cancers mostly diagnosed in females, and the second most frequent malignancy-associated deaths worldwide, especially in the US and Asian countries [1, 2]. Approximately two million females are annually diagnosed and more than 620,000 deaths are newly recorded every year [3, 4]. Frequently, breast cancer (BC) is developed as a result of a genomic mutation. However, about 10% of BCs is inheritably come down from parents to their generations; whereas, more than 85% of BCs is developed in their lifetime [4, 5]. Inherited abnormalities in *TP53* and *PTEN* genes were studied to result in the high risk of malignant breast tumor progression [6, 7].

Gene expression profiling has recently played a critical role in medicinal selection for BC subtypes. The analysis of BC gene expression can be used for the molecular category of BC subtypes [8–10]. Two studies reported that this classification facilitates the determination of cure doses. The molecular subtypes of BC can be categorized into luminal-A, luminal-B (including HER2+ /−), HER2+, and triple-negative (TN) [11, 12]. These subtypes are pivotal for cure choice and are correlated to the biological characteristics of BC.

MicroRNA (miRNA or miR), which is a type of small non-coding RNAs (sncRNAs), is synthesized from eukaryotic genomes, consisting of a single-stranded RNA of about 19-22 nt in length [13, 14]. These short RNAs are described as regulators of coding (cRNAs) and sncRNAs in eukaryotic cells because they are involved in silencing and stabilizing targeted cRNAs [14, 15]. MiRNAs play also several regulatory roles in several cellular processes; cell development, proliferation, migration, invasion, and death [16]. Because more than 50% of RNA molecules has been detected to be controlled by miRNAs, these mRNAs were damaged because of the effect of aberrant miRNA in malignant cells.

MiRNAs in human malignant tumors were found to act as oncogenes or tumor suppressors. Oncogenic miRNAs in tumor progress play a negative role in stimulating genes that regulate cell development and the apoptosis process. Tumor suppressive miRNAs have a key role in silencing genes that modulate cell development and apoptosis [17, 18]. When normal cells do not undergo normal growth and apoptosis process, they normally cause tumor creation. Numerous recent experiments show that numerous miRNAs are directly implicated in modulating cell growth, proliferation as well as apoptosis [19, 20]. They play major roles in the pathogenesis of several human malignancies; such as breast, colorectal cancer, lung, leukemia liver, and brain [16, 18]. The miRNA expression level may be either down- or up-regulated in these cancers. Several molecular techniques, such as RNA sequencing, miRNA microarray, RT-PCR, and northern blot are applied to determine the expression level of them.

Numerous miRNAs have been recognized to be implicated in the pathogenesis of human breast cancer. It was found that the expression patterns of *miR-145*, *−125b*, *− 155*, and *-21*were significantly downregulated. In breast malignant cells, these miRNAs were observed to be associated with pathologic properties; cell proliferation, expression of progesterone, and estrogen receptors [21]. A recent study revealed that tumor-suppressive *miRNA-204-5p* plays a key role in targeting several oncogenic genes that are closely connected to BC pathogenesis [20]. Complete information on *miR-1275* expression level and its targets in BC have not been available; whereas, the expression profile of which has been analyzed in some human cancers. A study reported that down-expressed *miR-1275* leads to overexpression *claudin11* in cancer stem cells or tumor-initiating cells (CSCs/TICs) [22]. According to a study carried out on young women with BC, 6 miRNAs; including miR-1275, miR-1228*, miR-139, *miR-92b*, *miR-1207*, and *miR-3196,* were involved in the processes of cell movement, proliferation, and invasion [23]. It was also found that this miRNA was found to be downregulated in gastric cancer [24]. Another study found that *miR-1275* expression level was significantly abnormally deregulated in Ewing’s Sarcoma (ES) [25]. The significance of a large number of miRNAs has been reported to become an appropriate biomarker for human cancer diagnostics. However, the significance of *miR-1275* in BC is not reported. The objective of this study was to determine the expression level of *miR-1275* as a biomarker for BC diagnostics. Another objective is to identify the potential targeted genes of this miRNA.

## Material and method

### Collection of FFPE-blocks of BC samples

All procedures done in this research study involving human participants, human material, or human data were followed and approved by local Human Research Ethics Committee (HREC) at Science College in Salahaddin University-Erbil (Reference no. 4c/132). All methods while performing the study were also performed in accordance with the 1964 Helsinki Declaration and the written informed-consent and Permission for the publication were obtained from all research participants. FFPE-Block of 22 Kurdish cases with BC were collected at clinicopathological laboratories, called Al-Mufti and Luay. Any chemotherapy or radiotherapy was taken by none of these cases. For each patient, two paraffin blocks (NAT and BC tissue) were collected. We diagnosed the malignant and non-malignant areas. The non-malignant tissues were taken nearly 2 cm away from the malignant area. Clinical features of 22 cases were obtained using a questionnaire. The features were displayed in Table 1.

**Table 1:** Clinicopathological features of 22 cases with BC

Cases	Age	Tumor size	Stage	Lymph node metastasis	Lymphatic invasion	Venous invasion	E.R.	Pg.R.	Her2	Ki-67	Technique
Case1	74	4 cm	IIIA	Yes	1	1	Negative	Negative	Negative	60	MACE-seq & small RNA-seq
Case2	49	4 cm	IIIA	Yes	1	1	Negative	Negative	Negative	70	MACE-seq& small RNA-seq
Case3	35	4 cm	IIB	Yes	1	0	Negative	Negative	Positive	35	RT-PCR
Case4	64	4.5 cm	IIIA	Yes	1	0	Positive	Positive	Negative	5	RT-PCR
Case5	71	1.9 cm	I	No	0	0	Positive	Positive	Negative	Unavailable	RT-PCR
Case6	40	2.3 cm	IIB	Yes	1	0	Positive	Positive	Negative	4	RT-PCR
Case7	44	3.5 cm	IIIC	Yes	1	0	Positive	Positive	Negative	13	RT-PCR
Case8	53	2 cm	IIA	No	1	1	Positive	Negative	Positive	20–30	RT-PCR
Case9	46	1.8 cm	IIA	Yes	1	1	Positive	Negative	Positive	10	RT-PCR
Case10	33	1.4 cm	IIA	No	0	0	Positive	Positive	Positive	8	RT-PCR
Case11	49	5 cm	IIIC	Yes	1	0	Negative	Positive	Negative	13	RT-PCR
Case12	45	1.9 cm	IIIB	No	0	0	Negative	Negative	Negative	80–90	RT-PCR
Case13	64	1.5 cm	IIA	No	0	0	Negative	Negative	Negative	80–90	RT-PCR
Case14	49	1.7 cm	IIA	No	0	1	Negative	Negative	Negative	70–80	RT-PCR
Case15	35	1.3 cm	IIA	No	0	0	Negative	Negative	Negative	90	RT-PCR
Case16	52	3 cm	IIA	No	0	0	Negative	Negative	Negative	60	RT-PCR
Case17	18	2.5 cm	IIA	No	0	0	Positive	Negative	Negative	50	RT-PCR
Case18	63	1.5 cm	I	No	0	0	Negative	Negative	Positive	12	RT-PCR
Case19	19	2.5 cm	IIB	Yes	1	0	Positive	Positive	Negative	24	RT-PCR
Case20	30	1.3 cm	IIA	No	1	0	Positive	Positive	Negative	15–20	RT-PCR
Case21	45	1.5 cm	IIA	No	0	0	Positive	Positive	Negative	15–20	RT-PCR
Case22	44	6 cm	IIIC	Yes	0	0	Positive	Positive	Positive	7	RT-PCR

### Total RNA extraction and library preparation for MACE-seq and small RNA seq

MACE-sequencing for mRNA expression and small RNA sequencing for small RNA expression were performed for two cases 1 and 2 (Table 1). The practical part of this task was performed in GenXPro GmbH laboratory. The protocol of total RNA extraction, small RNA separation, and library preparation was prepared from GenXPro GmbH in Germany. For each sample, total RNA was prepared from 10 μm of FFPE tissues employing the MACE-seq kit (GenXPro GmbH, Frankfurt, Germany). This kit contains all reagents to separate the molecule of the messenger RNAs (mRNAs). Then TrueQuant small RNA kit was used to separate small RNAs (sRNAs); including miRANs, small nucleolar RNAs (snoRNAs), small interfering RNAs (siRNAs), small nuclear RNAs (snRNAs), PIWI-interacting RNAs (piRNAs). Massive Analysis of cDNA Ends (MACE)-seq technique was used for the analysis of mRNA expression (GenXPro GmbH, Frankfurt, Germany). In MACE-seq, a specific molecular barcode, known as TrueQuant barcode, was used to barcode each mRNA molecule before PCR steps. The large molecules of the total RNA were utilized for the construction of the MACE-libraries and the small molecules for the construction of sncRNA libraries.

Four MACE-libraries (two mRNA libraries and two sRNA libraries) were generated for 2 BC and 2 NAT samples. Poly-adenylated mRNA was purified from the large fraction of total RNA using MACE-Kit (GenXPro GmbH, Frankfurt, Germany). First and second-strand cDNA was synthesized using the SuperScript® III First-Strand Synthesis System (GenXPro GmbH, Frankfurt, Germany). Then the cDNA products were attached to barcoded poly-T adapters. These barcodes are appropriate to attach to the MACE-seq flow well. The cDNA cap structures were specifically biotinylated. The full-length cDNAs which had biotinylated cap are attracted by streptavidin beads and that were randomly sheared to be about 300 bps, GenXPro’s TrueQuant barcodes were ligated. Four barcoded samples were sequenced simultaneously in one lane of an Illumina Hiseq2000 with 1 × 100 bps. To prepare miRNA libraries, small RNAs (16–25 bps) were chosen from the entire RNA molecules utilizing the flashPAGE™ Fractionator System (Life Technologies). TrueQuant Adapters were directly attached to the small RNAs, basically as mentioned by Hafner and his colleagues [26]. A Hiseq2000 machine (Illumina) was used to sequence the miRNA group comprising p5 and p7 adapters. The GenXPro GmbH prepared MACE and sRNA libraries. For removing flanking adapters, the organized reads were cut out for high-quality sequences. Bowtie 2 tool was later used for aligning the sorted reads to the nominated reference sequences and annotating with corresponding properties [27]. HT-seq tool was used to execute the quantification of mapped reads to each gene [28]. DESeq2 was used to execute differential expression analysis [29], and also is based on negative binomial generalized linear models. Results were compiled into a final table including significance parameters; including *P*.value, FDR and log2Fold-Changes. R-scripts were used to perfume Final Data visualization of the significantly expressed gene and the down/over-expressed genes.

### Total RNA extraction and cDNA synthesis for *miR-1275*

From the TrueQuant results, *miR-1275* was selected to confirm its expression from 20 Kurdish cases using Real Time-quantitative polymerase chain reaction (RT-qPCR) technique. The practical part of this task was performed in Salahuddin University Research Center (SURC). Forty block specimens (20 NAT and 20 BC tissues) were used for analysis of differential expression of *miR-1275*. For each experiment, 10 μm of the FFPE section was put in the microcentrifuge tube. Deparaffinization of the tissue was performed by incubation in 1 ml of xylene for 5 min at 50 °C. After incubation, the tissue was centrifugated to produce pellets at 14,000 rpm for 2 min. Then, the deparaffinized pellet was rehydrated with 1 ml of absolute ethanol. The tissue was centrifugated at 14,000 rpm for 2 min. The rehydration and centrifugation were repeated again. The tissue pellet was air-dried for 20 at room temperature. Then, the tissue pellet was homogenized using a PowerGen 125 Tissue Homogenizer. The total RNA molecules including miRNA were extracted using FFPE RNA/DNA Purification Plus kit (Cat. No. 54300, NORGEN BIOTEK CORP, Canada). Complementary DNA (cDNA) was synthesized using the miRNA All-In-One cDNA Synthesis Kit (Cat. No. G898, abmgood company, US).

### Determination of differential expression of *miR-1275* using RT-qPCR

All the items of RT-qPCR technique were ordered from abmgood company, US. RT-qPCR applications were performed to amplify the targeted miRNA among total cDNA molecules. For each solution well, the total volume was 20 μl which included 10 μl of BrightGreen miRNA qPCR MasterMix-ROX (Cat. No. MasterMix-mR), 0.5 μl for each forward and reverse primers (Cat. No. MPH01104), 1.4 μl of template cDNA, and 7.6 μl of nuclease-free water. In this study, two Universal/housekeeping miRNA primers were used, which represented the U6–2 primers (Cat. No. MPH0001) and SNORD44 primers (Cat. No. MPH0003). qPCR reaction was performed setting up the following 3-step cycling program. Enzyme activation was at 95 °C for 10 min, 40 cycles were set up for denaturing at 95 °C for 10 s, annealing at 63 °C for 20 s and extension at 72 °C for 20 s.

### Most common putative targeted genes regulated by *miR-1275*

In this study, 11 databases were searched for finding the most common putative targets of *miR-1275* (Table 2). Six putative targets (*DVL3, PPP2R2D, THSD4, CREB1, SYT7,* and *PRKACA*) were determined to possess binding sequence to *miR-1275.* Then differential expression of them was identified in the MACE-seq findings. GraphPad Prism, Version 8.0.1, was used to reveal the differential expression of these selected target genes.

**Table 2:** Brief information on target predicted databases was shown to find putative targets possessing binding sequence to *miR-1275*

Target predicted sites	Species	Tool properties	Website Websites
miRTarBase	Human, Mouse, Rat	Conservation, seed location	http://mirtarbase.mbc.nctu.edu.tw/php/index.php
Target scan	Human, Mouse, Fly, Fish, and Worm	Conservation, seed location	http://www.targetscan.org/
TargetMiner	Human, Mouse, Rat, Fly	Conservation, seed location	https://www.isical.ac.in/~bioinfo_miu/TargetMiner.html
MirTar2	Human, Mouse, rat, Dog and Chicken	Conservation, seed location	http://www.mirdb.org/
DIANA	Any	Conservation, seed match, and free energy	http://www.microrna.gr/microT-CDS
miRWalk	Human, Mouse, and Rat	Conservation, seed match and free energy	http://mirwalk.uni-hd.de/
miRmap	Human, Chimpanzee, Mouse, Rat, Cow, Chicken, Zebrafish, and Opossum	Conservation, seed match, and free energy	https://mirmap.ezlab.org/
RNA22	Human, Fruit Fly, Mouse, and Worm	Seed match and free energy	https://cm.jefferson.edu/rna22/
PicTar - Tools4miRs	Human, Mouse, Rat, Fly	Conservation, seed location	https://tools4mirs.org/software/target_prediction/pictar/
mirPath	Human, Mouse, D. melanogaster, C. elegans, R. norvegicus, D. rerio and G. gallus	Conservation, seed match and free energy	http://snf-515788.vm.okeanos.grnet.gr/index.php?r=mirpath/geneList
Microrna. org	Human, mouse, Fruit Fly, and rat	Conservation, seed match, free energy	http://www.microrna.org/

### Gene enrichment analysis of gene ontology for target genes of hsa-miR-1275

To study the biological role of hsa-miR-1275 in the BC, the online tool GenXpro (http://tools.genxpro.net/modules/GO Enrichment Tool v2) was applied to execute for the predicted target genes of miR-1275 to determine its function. The gene enrichment analyses of Gene Ontology (GO) include Cellular Component (CC), Biology Process (BP), and Molecular Function (MF).

### Analysis of clinicopathological data associated with BC

Association between *miR-1275* and its target genes was computationally analyzed to determine the clinical significance using databases of cBioPortal (http://www.cbioportal.org/) and OncoLnc (http://www.oncolnc.org/). Clinical data and expression levels of the *miR-1275* and its target genes were gained from these sites and then were downloaded on 10 September 2020. Then overall survival (OS) curve per day was designed using R software. The data and R scripts will be stored for future studies.

## Result

### Construction of expression profile of sRNAs for BC by TrueQuant technique

**Fig. 1:**
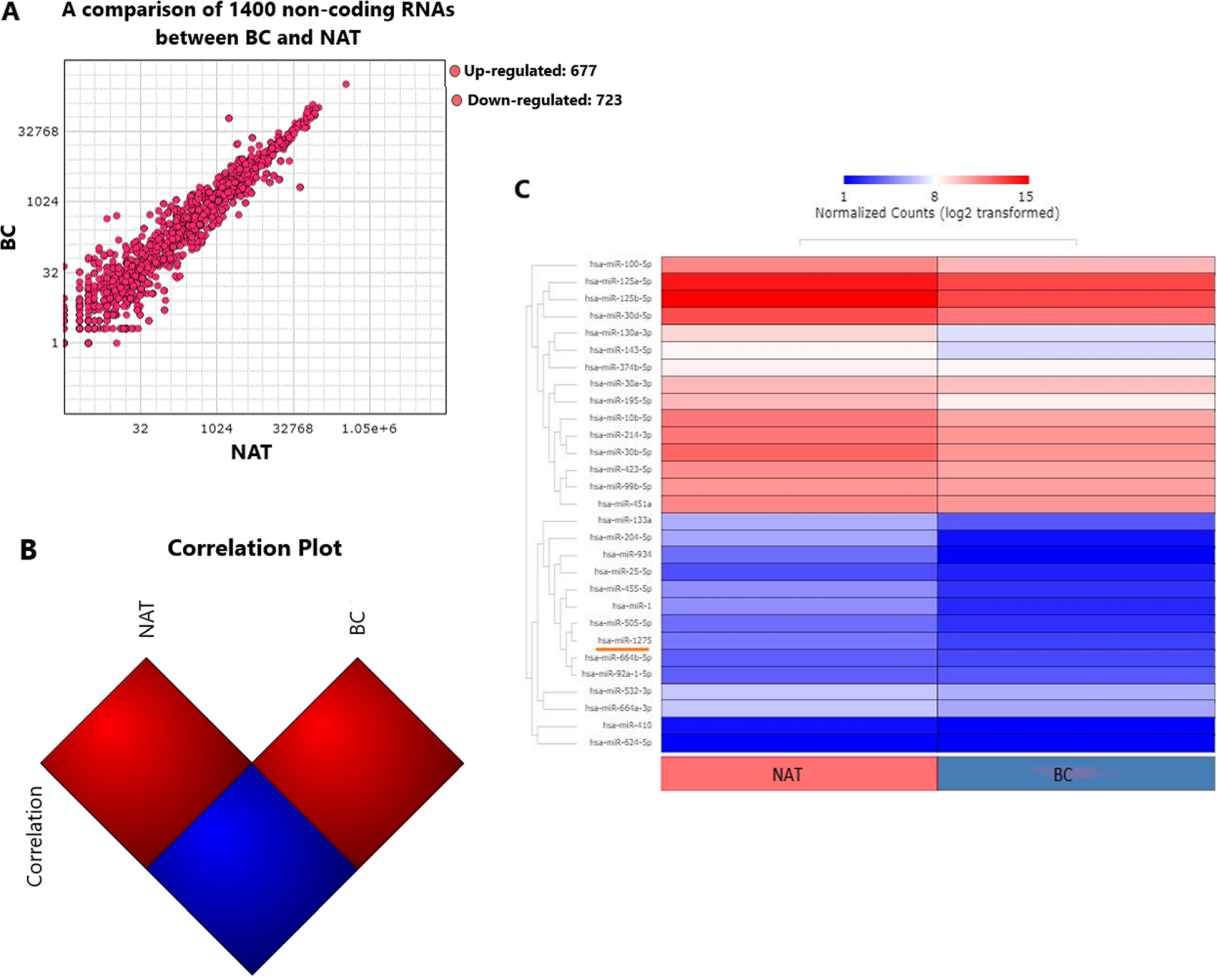
Differential expression analysis of non-coding RNAs by MACE-sequencing. **a** the expression profile of 1400 sRNAs in BC compared to NAT is plotted. Red dots represent the sample sRNAs. A light green dot represents *miR-1275* expression level in BC tissue compared to NAT. **b** Heat-map based clustering of several downregulated miRNAs in BC compared to NAT. *MiR-1275* is underlined. **c** correlation between BC and NAT in differential expression of sRNAs

By comparing sRNA expression profiles of BC and NAT, 1400 sRNAs were filtered out by SAM software. The raw data were then standardized and log2-transformed to show on a scatter plot (Fig. 1a). Among 1400 sRNAs, 723 non-coding RNAs were downregulated, which are located on the right and lower side of the trumpet plot but 677 sRNAs were upregulated, which are on the left and upper side of the trumpet. Each dot on the scatter plot represents the sRNA. Among 1400 sRNAs, 520 miRNAs were differentially expressed. 185 miRNAs were down-expressed, but 335 miRNAs were overexpressed. The x-axis denotes the data of the NAT and the y-axis denotes the data of the BC. A correlation plot was then constructed to show expression levels of sRNAs between BC and NAT (Fig. 1b). Blue color denoted the correlation of sRNAs between the BC and NAT but red color denoted the misrelation of sRNAs. However, a Heat map was designed to show 29 miRNAs which were markedly downregulated in BC compared to NAT (Fig. 1c). Moreover, the Limma package in R language was utilized to searching differentially expressed ncRNAs (DEncRNAs) between BC and NAT. Bonferroni in the multtest package was applied to adjust the *P.* value into the FDR. The FDR < 0.05 and |log2 FC| > 0.5 were applied as the cutoff criteria for the DEncRNAs. Table 3 shows the information on the accession number, *P.* value, genome loci, Log2 fold change (Log2FC), and False Discovering Rate (FDR) of these 29 miRNAs. In this study, *has*-*miR-1275*, which was underlined with red color in the heat map, was focused to identify expression level and sequence.

**Table 3:** Comparison of marked down-expressed miRNAs in BC with NAT

miRNA	miRBase accession	Location	Log2FC	*P*. value	FDR
*Hsa-miR-1*	*MIMAT0031892*	20q13.33	−2.6291	0.3109	1.0
*Hsa-miR-100-5p*	*MIMAT0004512*	11q24.1	−1.2349	0.2824	1.0
*Hsa-miR-10b-5p*	*MIMAT0000254*	2q31.1	−1.2852	0.261	1.0
*Hsa-miR-125a-5p*	*MIMAT0000443*	19q13.41	−1.4153	0.213	1.0
*Hsa-miR-125b-5p*	*MIMAT0000423*	11q24.1	−2.0041	0.083	1.0
*Hsa-miR-1275*	*MIMAT0005929*	6p21.31	−1.4150	0.6 14	1.0
*Hsa-miR-130a-3p*	*MIMAT0004593*	11q12.1	−2.0168	0.104	1.0
*Hsa-miR-133a-5p*	*MIMAT0026478*	18q11.2	−2.3885	0.220	1.0
*Hsa-miR-143-5p*	*MIMAT0004599*	5q32	−1.2823	0.308	1.0
*Hsa-miR-204-5p*	*MIMAT0000265*	9q21.12	−4.0627	0.086	1.0
*Hsa-miR-21-3p*	*MIMAT0004494*	17q23.1	−0.0365	0.995	1.0
*Hsa-miR-214-3p*	*MIMAT0000271*	1q24.3	−0.8746	0.440	1.0
*Hsa-miR-25-5p*	*MIMAT0004498*	7q22.1	−1.2630	0.793	1.0
*Hsa-miR-30a-3p*	*MIMAT0000088*	6q13	−0.2358	0.838	1.0
*Hsa-miR-30b-5p*	MIMAT0000420	8q24.22	−1.3254	0.245	1.0
*Hsa-miR-30d-5p*	*MIMAT0000245*	8q24.22	−0.9730	0.389	1.0
*Hsa-miR-374b-5p*	*MIMAT0004955*	Xq13.2	−0.2420	0.8435	1.0
*Hsa-miR-410-5p*	*MIMAT0026558*	14q32.31	−0.6780	0.989	1.0
*Hsa-miR-423-5p*	*MIMAT0004748*	17q11.2	−0.5727	0.614	1.0
*Hsa-miR-451a*	*MIMAT0001631*	17q11.2	−0.41900	0.711	1.0
*Hsa-miR-455-5p*	*MIMAT0003150*	9q32	−2.5081	0.305	1.0
*Hsa-miR-505-5p*	*MIMAT0004776*	Xq27.1	−1.5956	0.611	1.0
*Hsa-miR-532-3p*	*MIMAT0004780*	Xp11.23	−0.7496	0.612	1.0
*Hsa-miR-624-5p*	*MIMAT0003293*	14q12	−0.0931	1.0	1.0
*Hsa-miR-664a-3p*	*MIMAT0005949*	1q41	−0.9249	0.539	1.0
*Hsa-miR-664b-5p*	*MIMAT0022271*	Xq28	−0.5081	0.899	1.0
*Hsa-miR-92a-1-5p*	*MIMAT0004507*	13q31.3	−0.2085	0.972	1.0
*Hsa-miR-934*	*MIMAT0004977*	Xq26.3	−3.0931	0.417	1.0
*Hsa-miR-99b-5p*	*MIMAT0000689*	19q13.41	−0.1667	0.883	1.0

*Log2 FC* log2 fold-change, *FDR* False Discovering Rate

### Confirmation of *miR-1275* expression level by RT-qPCR

**Fig. 2:**
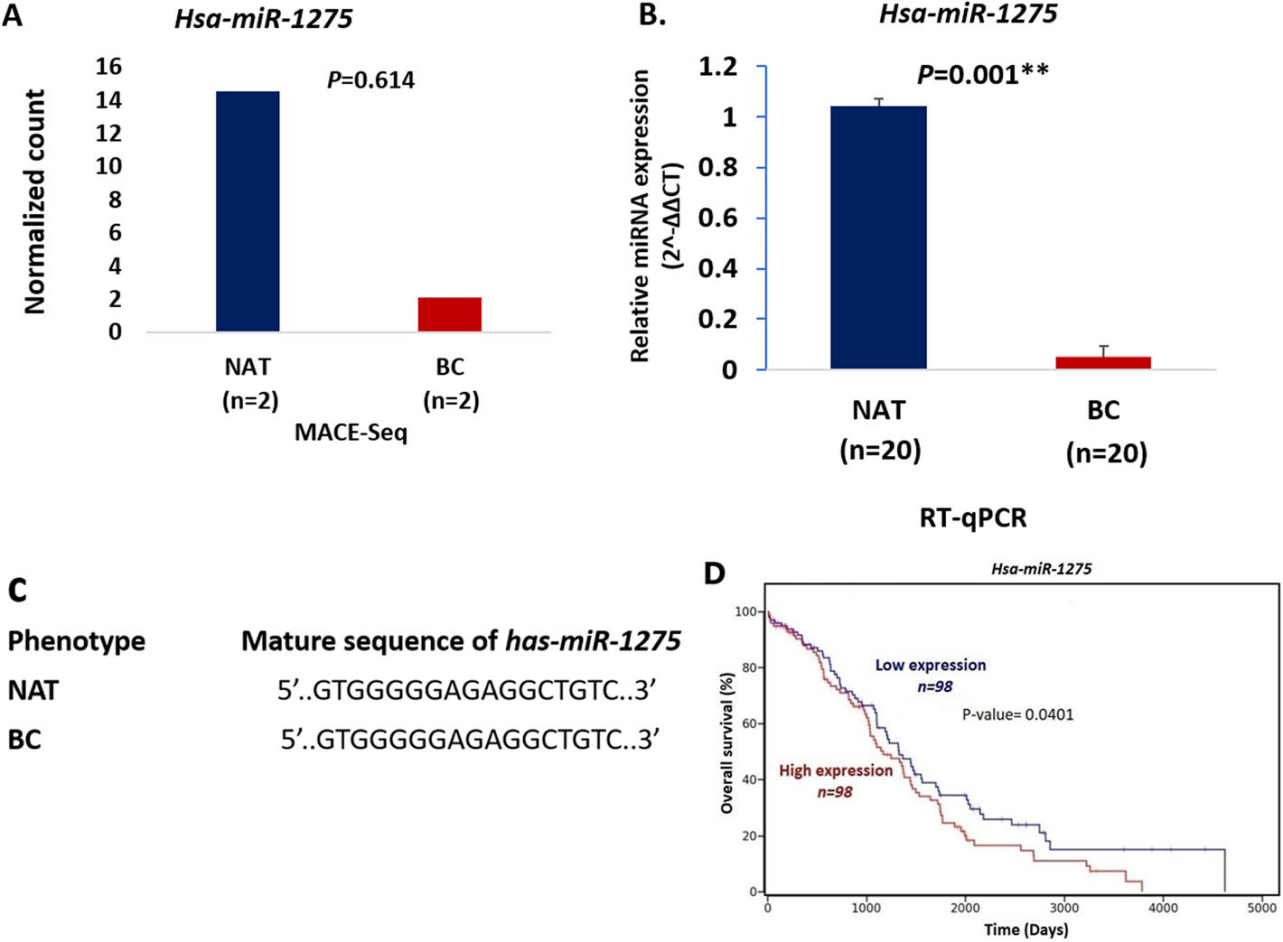
**a** Comparison of differential expression of *miR-1275* in BC compared to NAT using MACE-seq method. **b** Comparison of differential expression of *miR-1275* in BC compared to NAT using RT-qPCR method. **c** mature sequence of *miR-1275* in NAT and BC was shown to be identical. **d** Kaplan–Meier overall survival curve designed to show the differential expression of *miR-1275* related to overall survival in the patients with the BC

In small RNA sequencing findings, the *miR-1275* expression level in 2 cases with BC was observed to be significantly downregulated compared to NAT. The *p.*value of which was 0.614 (Fig. 2a). Then, this result was confirmed using an RT-qPCR machine in 20 other cases with BC. The *miR-1275* expression level was detected to be markedly decreased in BC compared to NAT and the *P*-value of this was 0.001 (Fig. 2b). The mature sequence of *miR-1275* in the BC and NAT was sequenced and made up of 17 nucleotides (10G, 3 T, 2C, and 2A). The mature sequence in both is also identical (Fig. 2c). However, Kaplan–Meier overall survival curve was designed to show the effect of the *miR-1275* expression on the prognosis of cases with BC. Data of overall survival (OS) was gained from The Cancer Genome Atlas (TCGA) database and analyzed by the R program. OS curve displayed that cases were separated into two classes according to *miR-1275* expression. The decreased *miR-1275* (*P*-value = 0.0401) was related to overall survival in cases with BC (Fig. 2d).

### Comparison of differential expression of genes in BC with NAT tissues

**Fig. 3:**
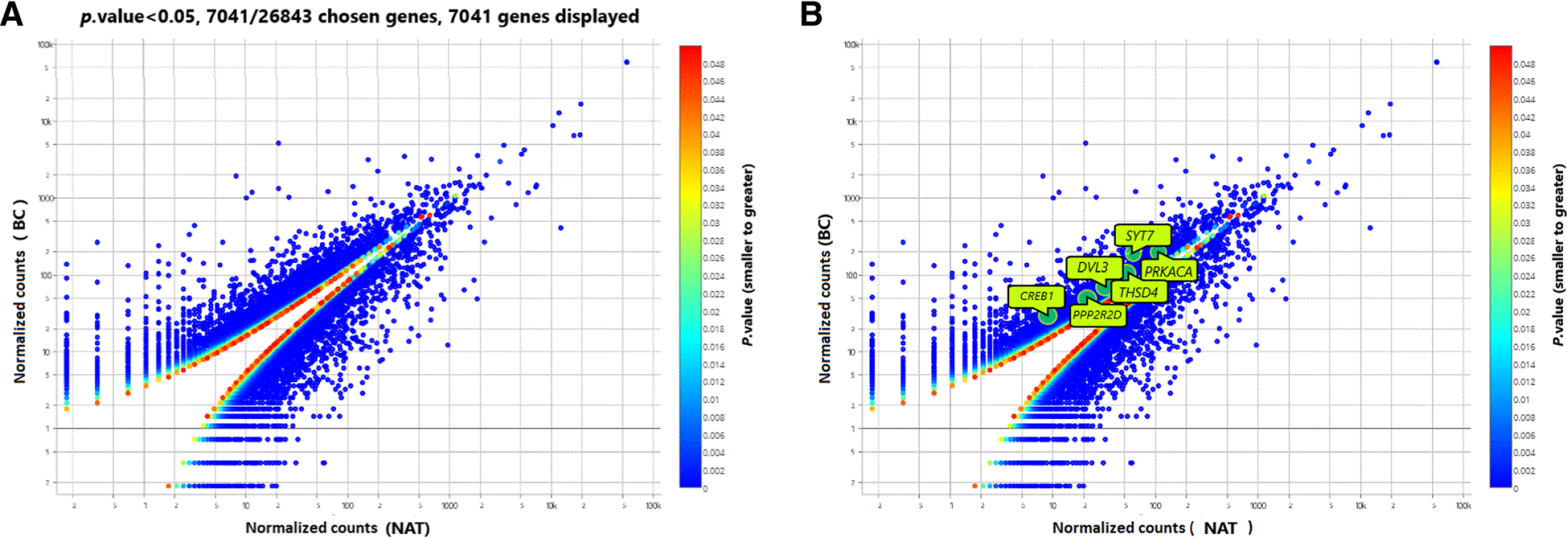
**a** Scatter plot analysis of gene expression profile displays up- or down-regulation of genes in BC tissue, compared to NAT. Each point denotes the average value of one transcript in the experiment. The expression difference is taken account of significance for a *P.* value (0.05). **b** Outlined points and names denote the selected target genes for *miR-1275*

In MACE-sequencing results, differentially expressed genes in two cases with BC were compared with NAT tissues (Fig. 3a). Twenty-six thousand eight hundred forty-three differentially expressed genes (*P* ≤ 0.05) were filtered out by SAM software. In order to show genes that were more significantly different in their expressions, 7041 genes were standardized and log2-transformed to show on a scatter plot. Three thousand six hundred twenty-four genes of which were significantly overexpressed and 3417 genes were significantly down-expressed in BC compared to NAT. The *P.* value for that was ranged from smaller (Blue) to greater (Red). Each point on the scatter plot represents the gene. The x-axis denotes the data of the NAT and the y-axis denotes the data of the cancerous tissue.

### Candidate target genes regulated in BC by *miR-1275*

**Fig. 4:**
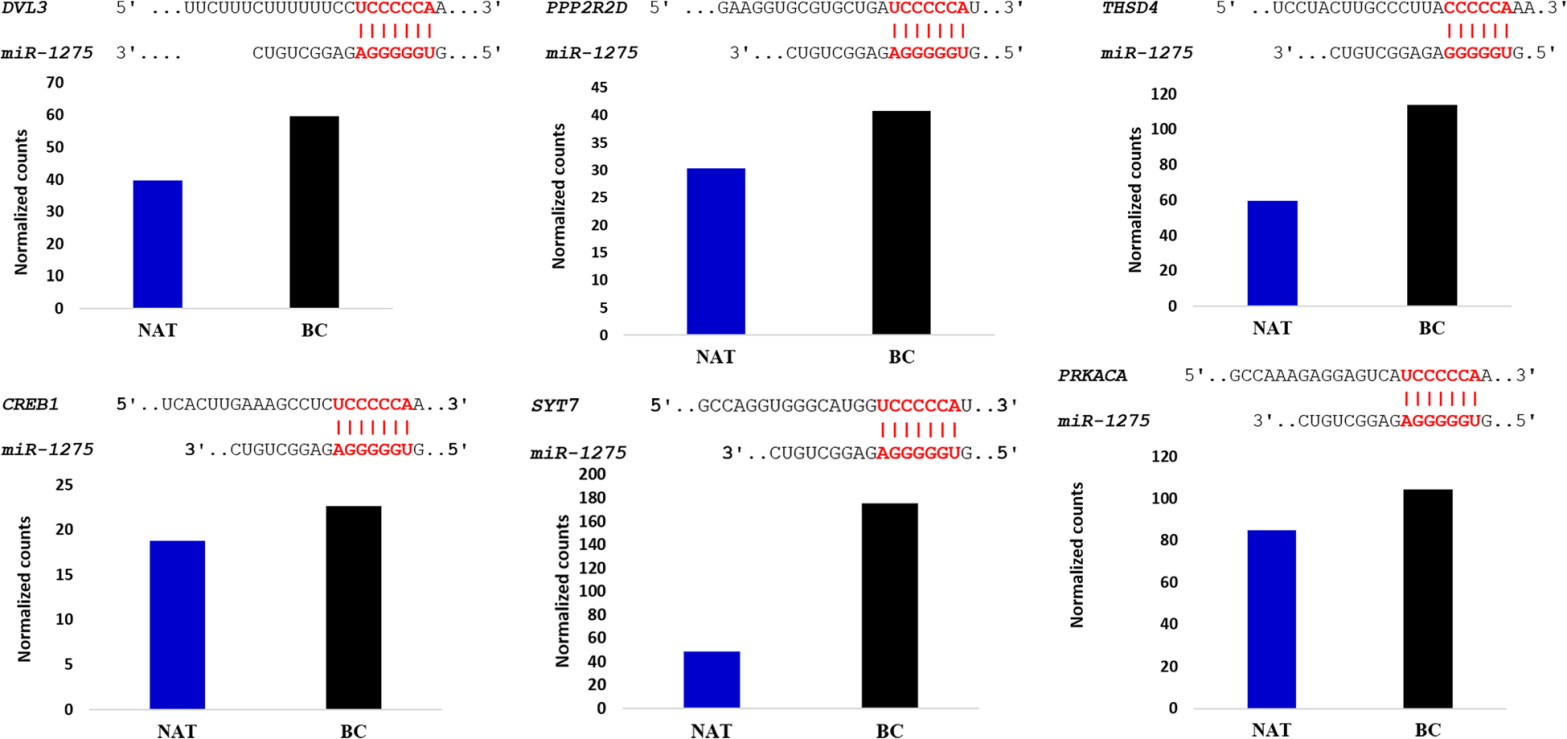
Target genes and *miR-1275* are combined in the seed region, including 6 to 8 nucleotides in the 5′ end, showing in red color. Differential expression level of target genes in two cases with BC compared to NAT

More than ten computational prediction programs were utilized for discovering the strongest candidate genes possessed *miR-1275* binding sites in the 3′ -UTR. Six predicted genes (*DVL3*, *PPP2R2D, THSD4, CREB1, SYT7,* and *PRKACA*) were selected to have the binding sites to *miR-1275* (red bonds and bps between targets and *miR-1275* in Fig. 4). The information on these six predicted genes was summarized in Table 4. Eleven databases showed that *DVL3* and *PPP2R2D* possessed the binding site to *miR-1275*; whereas, *THSD4*, and *CREB1* were confirmed in ten prediction programs to be targeted by *miR-1275,* but *SYT7* and *PRKACA* were confirmed by six tools to be predicted targets. These putative target genes are important for biological analysis of the BC tissues because the over-or down-expression of which can play a damaging role in several cellular processes and contribute to cancer progression and tumorigenesis.

**Table 4:** Candidate target genes possessing binding sequence to *miR-1275* was shown

Target gene	Ensemble ID	Position on chromosome	No. of sites predicted the gene as *miR-1275* target
*DVL3*	ENST00000313143.3	3q27.1	11
*PPP2R2D*	ENST00000422256.2	10q26	11
*THSD4*	ENST00000355327.3	15q23	10
*CREB1*	ENST00000432329.2	2q34	10
*SYT7*	ENST00000263846.4	11q12.2	6
*PRKACA*	ENST00000308677.4	17q24.2	6

### Determination of expression level of candidate target genes in MACE-seq findings

In MACE-seq finding, the six predicted genes (*DVL3, PPP2R2D, THSD4, CREB1, SYT7,* and *PRKACA*) were pointed and named in the BC cells as compared to NAT (Fig. 3b). Then the differential expression of which and their binding sites to *has-miR-1275* were shown in Fig. 4. Among the targets, the *SYT7* gene was more overexpressed in BC, as compared to the *PRKACA* gene. The overexpression level of the *THSD4* gene was also higher than the up-regulation of *PPP2R2D* and *DVL3* genes. *CREB1* was upregulated but more over-expressed than the *ST73* gene. Table 5 demonstrates the information on *P.*value, False Discovering Rate (FDR), and Fold Change (FC) of these six predicted genes possessed *miR-1275* binding sites in the 3′ -UTR. They were identified as potentially modulated by *miR-*1275 using computational prediction databases and TCGA algorithm.

**Table 5:** Experimentally validated target genes of *miR-1275* in BC

Targets	Gen ID	Description	*P*.value	FDR	Log2fc
*DVL3*	ENSG00000161202	Dishevelled segment polarity protein 3	6.98E-04	3.48E-03	0.58975
*PPP2R2D*	ENSG00000175470	Protein phosphatase 2, regulatory subunit B, delta	1.53E-03	6.95E-03	0.616981
*THSD4*	ENSG00000187720	Thrombospondin type 1 domain containing 4	2.28E-12	3.63E-11	0.938081
*CREB1*	ENSG00000118260	cAMP responsive element binding protein 1	3.12E-01	5.13E-01	0.269971
*SYT7*	ENSG00000011347	Synaptotagmin 7	1.28E-48	8.14E-47	1.84905
*PRKACA*	ENSG00000072062	Protein kinase cAMP-activated catalytic subunit alpha	1.77E-02	5.68E-02	0.296759

**Fig. 5:**
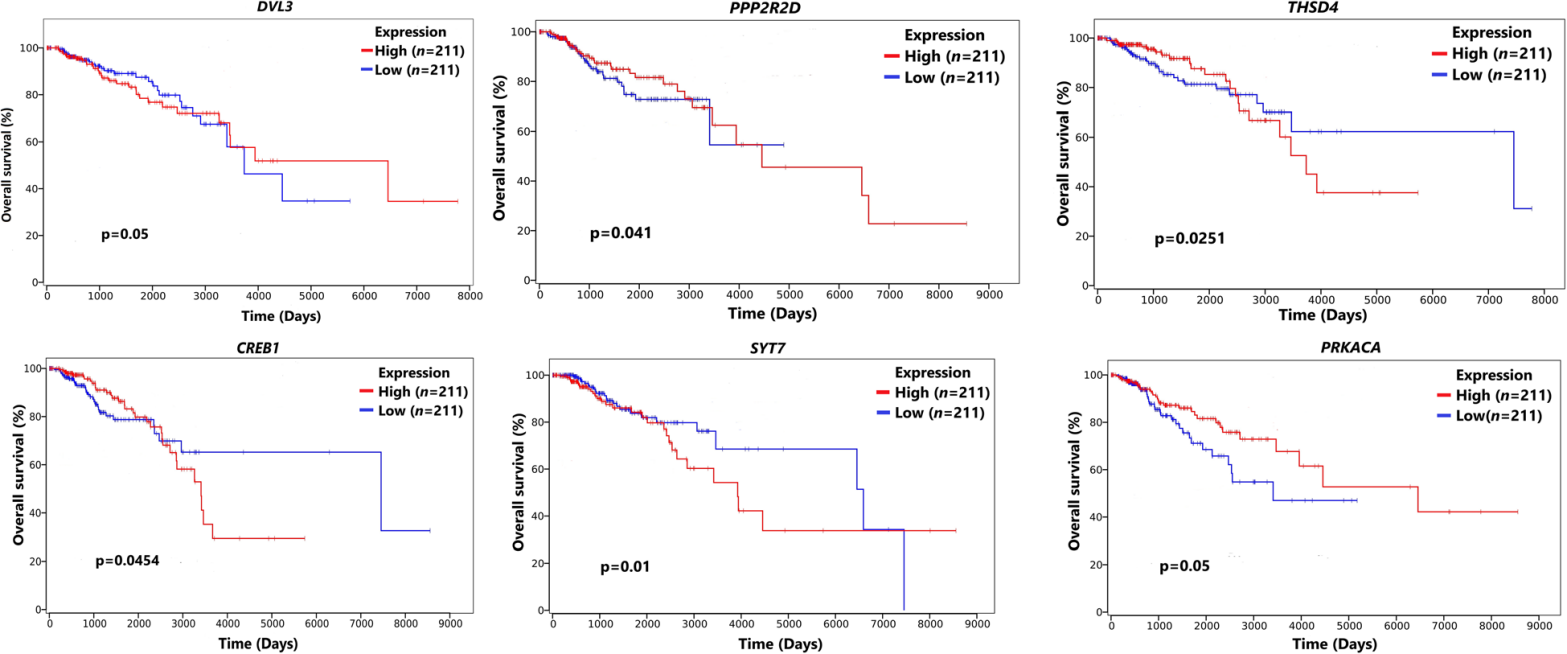
An association between the expression levels of six genes (*DVL3*, *PPP2R2D*, *TSHD4*, *CREB1*, *SYT7*, and *PRKACA*) and histopathological significance was shown using data from TCGA database. The Kaplan–Meier overall survival curves show that patients with BC were separated into 2 classes according to their expression levels

However, the relationship between the expression level of these 6 candidates and histopathological significance was analyzed based on data from the TCGA database. Among 204 target genes, *DVL3*: *P* = 6.98E-04*, PPP2R2D*: *P* = 1.53E-03*, THSD4*: *P* = 2.28E-12*, CREB1*: *P* = 3.12E-01*, SYT7*: *P* = 1.28E-48*,* and *PRKACA*: *P* = 5.68E-02 were markedly relationship with worse prognosis in cases with BC. For all targets, the Kaplan Meier curve was designed to show the correlation between OS and gene expression in cases (Fig. 5).

### Enrichment analysis of gene ontology (GO)

**Fig. 6:**
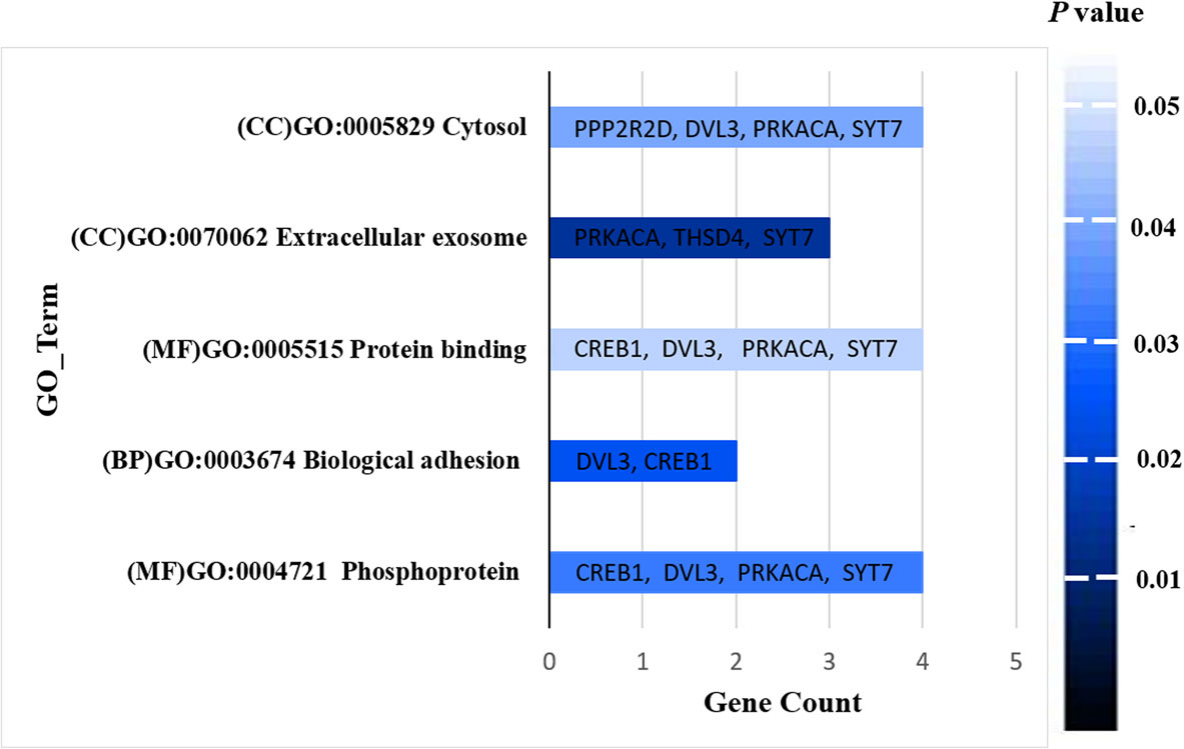
The gene ontology functional enrichment analyses of differentially expressed genes (DEGs) for targeted genes of hsa-miR-1275

Based on the GenXpro databases, GO enrichment analysis of differentially expressed genes (DEGs) was carried out for determination of the biological role of the target genes of hsa-miR-1275 in the BC. The up-regulated DEG of *DVL3,* PPP*2R2D, THSD4, CREB1, SYT7,* and *PRKACA* was enriched in the GO_terms, such as the extracellular exosome (GO:0070062; *P* = 0.014527), cytosol (GO:0005829; *P* = 0.041234), phosphoprotein (GO:0004721; *P* = 0.036901) (Fig. 6).

### The role of *miR-1275* by targeting selected putative genes in BC

**Fig. 7:**
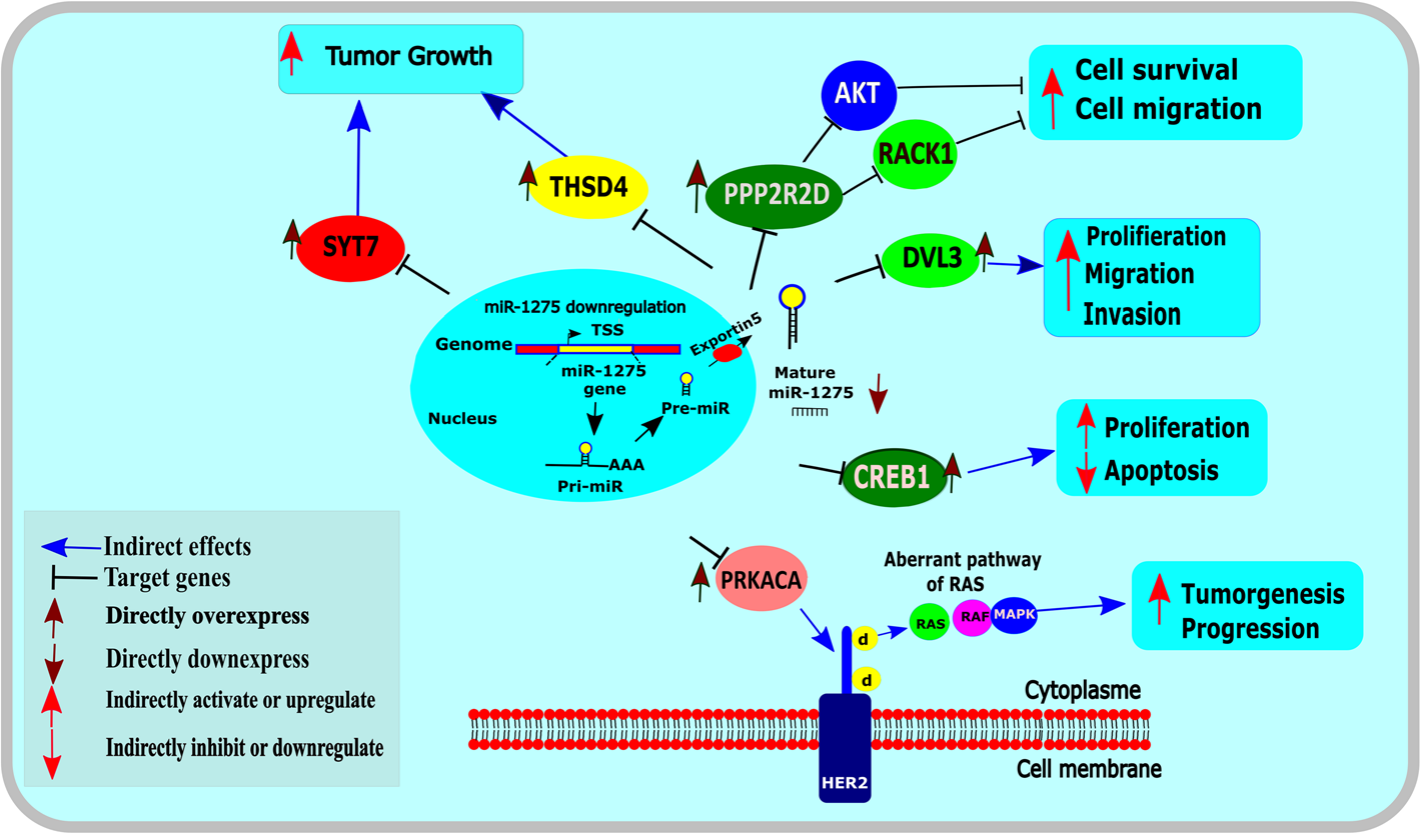
*miR-1275* putative targets and their roles in the BC

MiRNAs are implicated in silencing mRNA transcripts through matching or mismatching with target mRNAs. As hypothesis of microRNA biogenesis, significant strands of *miR-1275* come from miRNA duplex are joined into the RISC protein and modulate the mRNA transcripts, but minor strands are broken down and cannot modulate gene expression. Figure 7 shows the relationship between *miR-1275* and these target genes. *miR-1275* can play an essential role in regulating several biological mechanisms; including cell growth, migration, differentiation, proliferation, and apoptosis. In this study, *miR-1275* was observed to regulate six genes related to tumor development. In MACE-seq. Findings, these six genes (*DVL3,* PPP*2R2D, THSD4, CREB1, SYT7,* and *PRKACA*) were detected to be over-expressed in BC cells as compared with NAT. Downregulation of *miR-1275* in breast cancer promotes cancer cell proliferation, cell differentiation, tumor growth, invasion and migration and also inhibits apoptosis through these six genes. All targeted genes were negatively regulated by *miR-1275*. *PPP2R2D* acts as a tumor suppressor in the signaling pathway in BC. The overexpression of which decreases *AKT* and *RACK1* abilities. Then these regulators increase cell survival and migration. *DVL3* is implicated in the breast cancer pathways and negatively controlled by *miR-1275*. The up-regulation of which increases cancer cell proliferation, migration, and invasion*.* The cancer cell proliferation ability is also increased when *miR-1275* becomes overexpressed. Moreover, *CREB1* was found to reduce the apoptosis process and increase cell proliferation in breast cancer; whereas, *PRKACA* plays a key role in tumorigenesis and development of BC. However, the function of *THSD4* and *SYT7*, currently unidentified, may boost tumor growth in breast cancer.

## Discussion

There is evidence that a single microRNA modulates multiple protein-coding and non-coding genes in different ordinary cells and cancerous cells. In human cancer cells, new RNA groups can be detected using the specific nature of microRNA from relevant microRNA analysis. Multiple high-throughput approaches, such as DNA microarrays, MACE-sequencing, PCR-based arrays, and RNA-sequencing, are now available and have made microRNA expression profiles of BC, showing the irregular expression of numerous miRNAs [3, 30–32]. One approach to detect the most essential miRNA from numerous miRNAs is to detect differential expression of miRNAs which have been shown in numerous experiments. Several types of research have reported that multiple miRNAs, such as *miR-100, miR-107, miR-205-3p, miR-122* and *miR-99a-5p*, are continuously down-expressed and act as tumor-suppressive miRNA in BC cells [4, 30–35]. In this study, these miRNAs were found to be downregulated in tumor cells but only *miR-1275* was focused and their putative target genes were newly explored in paraffin-embedded BC tissues. Several types research have reported that this miRNA is down-expressed in gastric and nasopharyngeal carcinoma and function as a tumor suppressor [36–38]; whereas, this miRNA is overexpressed in non-small lung cell cancer, squamous carcinoma, and chronic myelogenous leukemia [39–41]. Some recent studies revealed that this downregulated miRNA was detected to have an essential effect on cancer cell proliferation, migration, invasion, metastasis, and angiogenesis through targeting multiple oncogenic genes *HOXB5*, *WNT7B* and *LncRNA* HAND2-AS1 [36, 38, 41]. One previous study showed that *miR-1275* regulates *IGF1, NFIX*, *Claudin11* in very young women with BC [23]. Whereas, down-expression of *miR-1275* in all subtypes of paraffin BC tissues was not fully investigated. In this study, down-expression of miRNA was observed in all subtypes of paraffin-embedded BC tissues of 21 cases of different ages.

After that, the *miR-1275*-modulated putative targets and their pathways were aimed to explain in the cells of BC. The expression of DVL3, PPP2R2D, TSHD4, CREB1, SYT7, and PRKACA were experimentally observed to be high in the cells of BC. Based on the databases of miRNA target prediction, they were selected and closely correlated with poor prognosis. The biological role of these genes was determined according to the GO terms enrichment with DEGs. These genes were involved in the GO terms of the cytosol, extracellular exosome, protein binding, phosphoprotein and biological adhesion. Two previous studies reported that the overexpression of which could induce a significant impact on the biological processes of the BC progression [42, 43]. Among these candidate genes, three genes (*PPP2R2D*, *DVL3*, and *CREB1*) were shown to be strongly targeted by the *miR-1275* in the BC cells. Studies showed that these regulators were found to reduce cell survival and migration in cancer cells [42, 43]. *DVL3* is observed to be implicated in the breast cancer pathways [36] and negatively regulated by *miR-1275*. The up-regulation of this gene can increase cancer cell proliferation, migration, and invasion in BC cells*.* The overexpression of which increases cancer cell proliferation ability [44]. Another target gene, *CREB1,* and *PRKACA* show also a negative correlation with *miR-1275* level*.* Whereas *CREB1* was found to reduce the apoptosis process and increase cell proliferation in breast cancer [45], *PRKACA* plays a key role in tumorigenesis and development of BC [46]. However, the role of *THSD4* and *SYT7*, currently unidentified, may enhance tumor growth in a variety of cancers, especially breast cancer [47, 48].

## Conclusion

In the present study, differential expression profiles of mRNA transcripts and sRNAs were analyzed in two cases with BC compared to NAT. Down expression of *miR-1275* was measured using the RT-qPCR technique. Down-expression of which develops breast cancer by increasing the activity of biological processes; such as growth, migration, invasion, and metastasis. Upregulated *miR-1275* prevented BC development by modulating the direct expression of *DVL3*, *PPP2R2D*, *TSHD4*, *CREB1*, *SYT7*, and *PRKACA*. This is the first study revealing that *miR-1275* function as a tumor-suppressive miRNA in BC cells, regulating numerous targets which were closely related with BC pathogenesis and oncogenesis.

## Data Availability

Although row data of MACE-sequencing and sRNA may be available in the database of GenXpro, at (https://genxpro.net/, available project: 20190618_Majed_MACE SMajed 2019-06-18 18:44:18.841903 + 00:00), These data will be further studied for another research in the future. The findings described in this manuscript were provided by the Co-author.

## Abbreviations

MiRNA: MicroRNA
FFPE: Formalin fixed paraffin embedded
BC: Breast cancer
NAT: Normal adjacent tissue
ncRNA: Non-coding RNA
sRNA: Small RNA
HREC: Human Research Ethics Committee
MACE: Massive Analysis of cDNA Ends
E.R.: Estrogen receptor
Pg. R.: Progesterone receptor
HER2: Human epidermal growth factor receptor 2
OS: Overall survival
TCGA: The Cancer Genome Atlas
FDR: False Discovering Rate
FC: Fold change
DEncRNA: Differentially expressed ncRNA
DGE: Differentially expressed gene
GO: Gene ontology
CC: Cellular compartment
MF: Molecular function
BP: Biological process
